# Effect of a pragmatic lifestyle modification intervention on physical activity levels and body mass index among obese and overweight adolescents in Udupi, India: a cluster randomized trial

**DOI:** 10.12688/f1000research.153483.1

**Published:** 2024-08-01

**Authors:** prateek srivastav, Vaishali K, H Vinod Bhat, Suzanne Broadbent

**Affiliations:** 1 Department of Physiotherapy, Manipal College of Health Professions, Manipal Academy of Higher Education, Manipal, Karnataka, 576104, India; 2 The Apollo University, Chittoor, Andhra Pradesh, 517127, India; 3 School of Health, University of Sunshine Coast, Sippy Downs, Queensland, 4556, Australia

**Keywords:** Adolescents, lifestyle, diet, physical activity, parents, health education, school

## Abstract

**Background:**

Determine the effects of a multifactorial lifestyle intervention on physical activity (PA), BMI and health-related quality of life (QoL) in obese and overweight adolescents.

**Methods:**

Nine schools in India were clustered randomly in a 12-month study with students allocated to a multifactorial intervention (MFI), or exercise only (EX) or control (CON) group. Participants were adolescents aged 11-16 years (n=671). In the MFI group, adolescents and their parents received lifestyle education using a validated booklet combined with a PA intervention for school students. The EX group received school-based PA only; the CON group continued regular activities. Primary outcomes were PA levels measured with the PAQ-A, and BMI; the secondary outcome was health-related QoL. A linear regression statistical model was used to analyse time, group effects and interactions, with Bonferroni correction for within-group differences at baseline (T0) and at 12-weeks (T1) (post-intervention), 6-month (T2) and 12-month (T3) follow-ups.

**Results:**

Significant time and group effects observed for all groups with PA scores (p<0.001), with MFI group having largest increase in PA; with BMI (p<0.001) and MFI showing the least gain in BMI; and HRQOL (p<0.001), with MFI group showing greatest improvement in scores. There were significant increases in PA at T1 and T3 time-points with the EX group, and at T3 time-point only for MFI and CON, with MFI group showing largest increase in HRQOL scores. BMI increased significantly for all groups at T2 (MFI p=0.001, EX p<0.001) and T3 (p<0.001), while HRQOL increased significantly for both MFI and EX at both follow-ups (p<0.001).

**Conclusions:**

School-based lifestyle MFI was more effective for improving PA, lifestyle behaviours and HRQOL than exercise alone for adolescents, although BMI was not reduced. MFI with PA could be an effective school-based approach for behaviour modification but BMI has limitations for measuring body composition changes.

**Registration:**

CTRI/2019/04/018834 (30/04/2019).

## 1. Introduction

Adolescent obesity is a global issue in developed as well as developing nations. ^1^^–^^3^ A 2020 study predicted that by 2030, the number of obese and overweight adolescents will exceed 250 million globally, with China, India and USA being largest contributors. ^4^ This has made weight gain a prevalent long term health issue among adolescents. ^4^ Research shows there is 70%-80% likelihood of an overweight adolescent developing adulthood obesity, making them prone for short and long term health challenges. ^5^^,^^6^ Therefore, effective weight management and prevention programs become crucial during adolescence. ^6^

Interventions targeting lifestyle modifications have been a significant focus and first step in clinical and public settings for addressing obesity among adolescents. ^5^ In developing countries with resource-limited settings, lifestyle interventions offer a practical approach to address adolescent obesity. ^7^ Such interventions emphasize the importance of reducing high calorie food intake, promoting healthy eating habits, minimizing sedentary behaviour, engaging in regular physical activity (PA) and implementing behavioural modifications to educate parents about sustaining a healthy lifestyle. ^8^^–^^12^ Studies shows that making changes to the school food environment, such as banning sugary drinks and increasing availability of fruits and vegetables, led to a significant decrease in adolescent obesity rates. ^13^^,^^14^ Research has backed strategies promoting nutritional education and focusing on healthy food behaviours to promote a well-rounded diet, aligning with dietary recommendations for adolescents. ^15^^–^^17^ Furthermore, PA interventions for adolescents which included exercise promotion and sedentary time reduction in school settings have been found to be effective in lowering Body Mass Index (BMI). ^8^^,^^18^^,^^19^ However, the literature indicates that interventions focusing on diet and PA individually have a lower impact compared to being used in combination. ^20^ Furthermore, irrespective of body weight, increasing PA, reducing sedentary time and improving dietary patterns have been associated with reduced illness and better health outcomes, ultimately leading to an improved quality of life. ^21^^–^^23^

Previous studies in India have primarily evaluated the effect of PA and dietary modifications separately using outcome measures such as anthropometry, food habits and changes in PA levels. ^24^^–^^26^ These separate interventions have been moderately effective in the short term but have not provided long term solutions to the adolescent obesity problem. There is also limited literature on the role played by familial factors such as increasing family awareness of the importance of improving PA levels and healthy eating, which can significantly contribute to adolescent health. ^27^ A study conducted by Nayak et al (2016) among Indian adolescents concluded that parental involvement in weight loss interventions through PA and dietary education, and long-term follow up, can be crucial. ^28^ The Indian government also has recommended the need for implementing a systematic approach to improving health and promoting weight loss through primary and secondary prevention strategies. ^29^ Further, these prevention strategies should be implemented through government programs, school-based PA interventions, and nutritional education initiatives. ^26^^,^^27^

This cluster RCT aims to compare the effectiveness of a 12-week culturally appropriate, school-based PA and exercise intervention to an exercise-only intervention or no intervention for obese (OB) and overweight (OW) adolescents. We hypothesize that while both the interventions may increase PA, the lifestyle-based intervention will lead to more significant improvements in PA, BMI and health-related quality of life (HRQOL) and will be more effective in the long term.

## 2. Methods

### 2.1 Ethical statement

The study was conducted as per the principles of the Declaration of Helsinki and in accordance with the medical research involving human subject act (WMO). Permissions to approach the schools was sought from the Office of the Deputy Director of Public Instructions (DDPI), Udupi, Karnataka, India and each individual school principal’s permission was sought. The participants and their parents provided their informed consent at the beginning of the study and complete procedures were explained verbally and by administering the participant information sheet.

### 2.2 Study design

This study is a part of larger cluster randomized control trial, conducted for 12 weeks (T1), ^30^ with follow-up assessments at6 (T2) and 12 months (T3) post-baseline. The study was based on the Theory of Planned Behaviour, which suggests that an individual’s perceptions can be changed by culturally sensitive knowledge, leading to an intention to change behaviour. ^31^ The schools were randomized into three blocks based on geographical locations, with three schools in each block. A computer-generated randomization sequence was created for each block. The allocation concealment for the principal investigator was ensured by the placing of the random sequences in opaque envelopes by a faculty member of Manipal Academy who was not involved in the project. The adolescents from each school were divided into three groups – a multifactorial intervention (MFI group), an exercise-only intervention (EX group) and no intervention (CON group). Detailed procedures have been published previously. ^30^

### 2.3 Inclusion criteria

The inclusion criteria for the study were (1) males and females aged 11–16 years, who were attending English medium schools in Karnataka; (2) a BMI higher than the 85th percentile on the standard Centres for Disease Control BMI-for- age growth charts, where OW is at or above the 85th percentile and below the 95th percentile and OB is at or above the 95th percentile ^32^; (3) Participants should pass health screening using Physical Activity Readiness for Everyone (PAR-Q+). ^33^

### 2.4 Exclusion criteria

The exclusion criteria were (1) medically-diagnosed obesity due underlying disease or medications; (2) undergoing psychiatric treatments; (3) inability to attend twice-weekly intervention sessions in schools; (4) declared unfit to participate by general practitioner or paediatrician; (5) participating in another structured exercise programme. If participants reported any mental issue such as anxiety or depression, related to study participation, they were offered counselling at school, local hospital or through social services.

### 2.5 Sample size calculation

The sample size was calculated based on an unpublished pilot study considering BMI Z-scores as a primary outcome measure. The level of significance (α) was adjusted for three comparisons and considered at 0.05/3 to be 2.59, with a power of 80%. The anticipated standard deviation of the outcome variable for the population would be 2.13 and minimum clinical difference was assumed to be 1.5. The intraclass correlation coefficient (ICC) was determined to be 0.3. The proposed total sample size was 600, with 200 adolescents in the three groups (MFI, EX, CON). The detailed study protocol has been published previously. ^30^

### 2.6 Outcome measures

Primary outcome measures

The level of (PA) of the adolescent participants was measured using the Physical Activity Questionnaire- Adolescent (PAQ-A), ^34^ and BMI-Z scores using standard procedures. ^35^

Secondary outcome measure

Health-related quality of life (HRQOL) was assessed using Quality of Life Teen Report.

(PedsQL ^TM^ 4.0). ^36^ This questionnaire assesses physical function, emotional wellbeing, social functioning, and school performance.

### 2.8 Intervention

The evaluations and interventions were conducted in schools by a physiotherapist. Baseline assessment was completed for all the adolescents regardless of randomized group. The MFI group received PA and dietary education, plus parental education on healthy lifestyles, at the beginning of the intervention. The education consisted of a validated education manual for adolescents and parents, developed by qualified healthcare professionals and based on standard guidelines, ^37^ which reinforced culturally-adapted, age-specific healthy diet and optimum PA recommendations. The manual was printed in both English and the local language (Kannada) with illustrations of PA recommendations, dietary guidelines (e.g. portion size) and recommendations for decreasing electronic device screen time for adolescents. The education manual validation process has been explained in the published protocol. ^30^

The MFI group was given exercises based on American College of Sports Medicine (ACSM) guidelines for 60 min, twice weekly during school physical education (PE) classes under the supervision of a physiotherapist and PE teacher. Each session included moderate to high-intensity aerobic and strengthening exercises, with guidance on intensity using the Pictorial Child Effort Rating Table (PCERT), based on heart rate and breathing. Aerobic exercises (e.g. running, jogging, brisk walking) and strengthening exercises (e.g. tug of war, body weight resistance exercises) were part of each session. In addition, PA and dietary educational sessions were held for the adolescents at the school, while similar sessions took place for parents during parent-teacher meetings at the beginning of the intervention. Post-intervention follow up was conducted at the school. Any adolescents missing sessions for two continuous weeks were considered for dropout.

The EX group were given exercise only during PE classes under the supervision of a physiotherapist and PE teacher, with no diet, PA and behavioural education. The CON adolescents were encouraged to continue their regular activities and diet, with no interventions. All three groups were reassessed (1) post-intervention block at T1 and (2) with follow-up contact at T2 and T3 post-intervention time points.

### 2.9 Statistical analysis

Statistical analysis was performed using Jamovi software version 2.3.4. The demographic data was analysed using descriptive statistics (mean±SD), and Shapiro-Wilk tests were employed to assess the data normality. A general linear model with repeated measures and Bonferroni post hoc corrections at a level of significance of 0.05 was used to compare means across three groups and at different time points (group effect, time effect and group x time effect). Cohen’s effect size (ES) was used to indicate the magnitude of change with 0.2 being small, 0.5 medium and 1.2 being large.

## 3. Results

**Figure 1.**
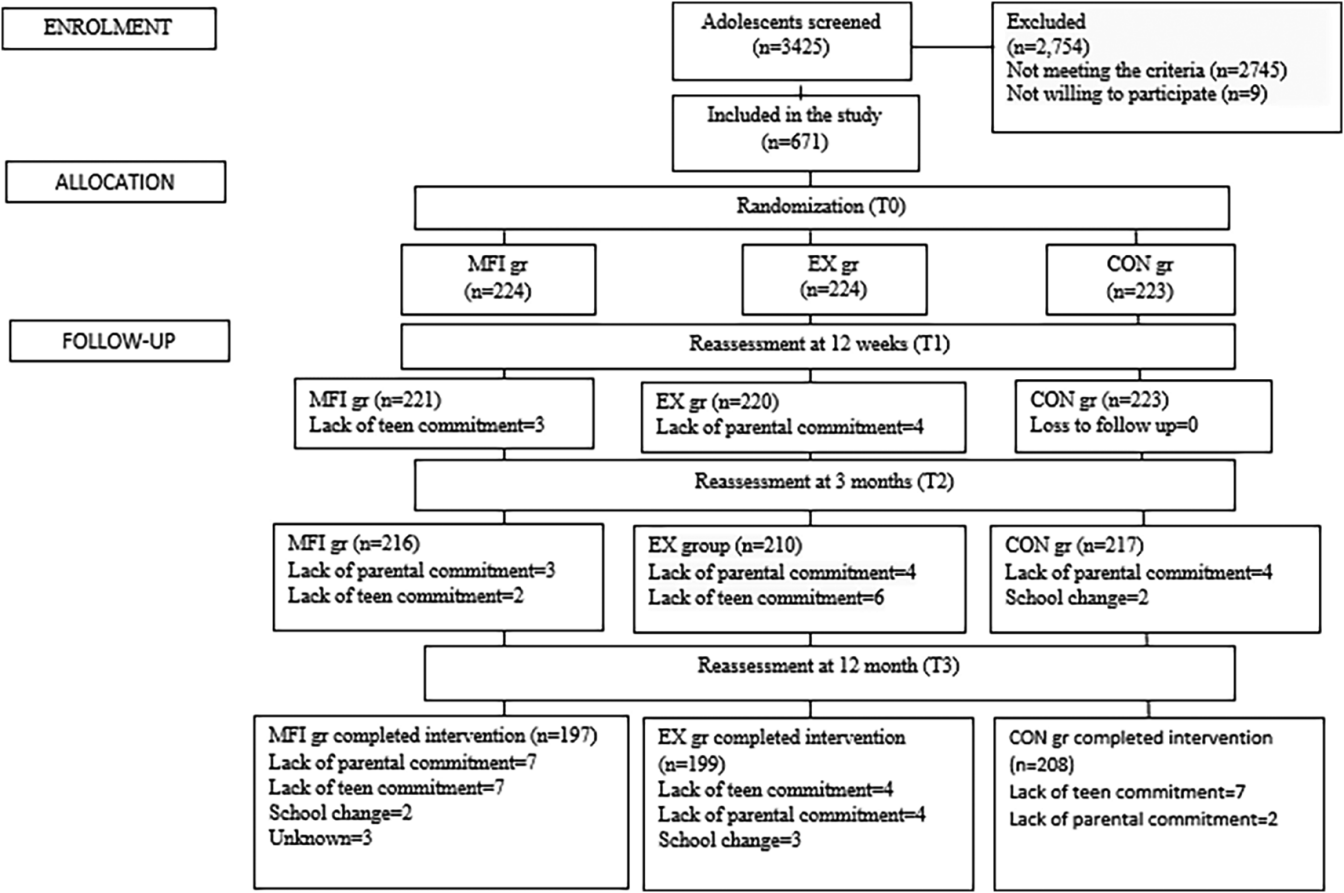
Flowchart of the study participants.

Out of a total of 3425 adolescents screened from nine schools, 671 obese (OB) and overweight (OW) adolescents participated in the study as per the inclusion criteria. These students were randomised into three groups with 224 in both the MFI and EX groups, and 223 in the CON group. After the follow-up period of 12 months, there were 197 adolescents in MFI group, 199 in EX group, and 208 in CON group due to dropouts. The CONSORT flowchart of the study is shown in Figure 1.

### 3.1 Participant’s baseline characteristics

The mean age of 126 (64%) males and 71 (36%) females in MFI group was 12.7±1.3 years, compared to 13.1±1.2 years for 121 (61%) males and 78 (39%) females in the EX group and 13.1±1.4 years for 136 (65%) males, 72 (35%) females in the CON group. The baseline demographic details of participants are shown in Table 1.

**Table 1.** Demographic details of the participants (shown in mean±SD and percentages).

Characteristics	MFI Group (n=197)		EX Group (n=199)		CON group (n=208)	
Age (yrs.)	12.7±1.3		13.1±1.2		13.1±1.4	
Gender	M=126 (64%)		M=121 (61%)		M=136 (65%)	
	F=71 (36%)		F=78 (39%)		F=72 (35%)	
Height (cm)	151.2+6.8		152.3+7.2		151.8±7.3	
Weight (Kgs)	53.0±6.8		54.0±6.9		53.2 ± 7.0	
Overweight (OW)	180 (91.4%)		185 (92.9%)		188 (90.4%)	
Obese (OB)	17 (8.6%)		14 (7.1%)		20 (9.6%)	
Outcomes	Mean±SD	p value	Mean±SD	p value	Mean±SD	p value
BMI (kg/m ^2^)	24.2± 2.17	<0.001	24.4±2.40	<0.001	24.6±2.52	<0.001
PA scores	3.92±0.56	<0.001	3.65± 0.38	<0.001	3.53±0.34	<0.001
HRQoL scores	60.6±10.9	<0.001	51.5±7.7	0.484	50.3± 7.75	0.332

### 3.2 Primary outcome analysis

Physical Activity

At T0, the mean PA scores were not significantly different (see Table 2). At T1, the score for the MFI group was 3.57±0.33, compared to the EX group with 3.50±0.31 and CON group with 3.43±0.35, At T3 the MFI group score was 4.55±0.32, while the EX and CON group changed to 3.91±0.33 and 3.60±0.33, respectively ( Table 2).

**Table 2.** Physical Activity (PAQ-A scores) of overweight and obese adolescent across groups and within-group difference.

Timepoints	MFI Group (n=197) Mean±SD	EX group (n=199) Mean±SD	CON group (n=208) Mean±SD	Time Effect(T) F: ES: p value	Group Effect(G) F: ES: p value	Group x Time effect F: ES: p value
T0	3.51±0.31	3.43±0.34	3.46±0.35	411.315 0.354 < 0.001*	374.027 0.199 < 0.001*	83.869 0.183 < 0.001*
T1	3.57±0.33	3.50±0.31	3.43±0.35			
T2	3.58± 0.34	3.58±0.33	3.48±0.31			
T3	4.55± 0.32	3.91± 0.33	3.6 ±0.33			
	MFI group		EX group		CON group	
Time-points	Diff	p value	Diff	p value	Diff	p value
T0-T1	-0.061	1.000	-0.067	1.000	-0.031	1.000
T0- T2	-0.070	1.000	-0.148	< 0.001*	-0.016	1.000
T0- T3	-1.045	< 0.001*	-0.482	<0 .001*	-0.215	< 0.001*

Analysis of the time and group effects ( Table 2) shows statistically significant changes in PA scores over all the time points within the study period (p<0.001), with a small to medium effect (ES=0.354). The overall group effect was also significant (p<0.001) with a small effect size (ES= 0.199). The group x time interaction was statistically significant (p<0.001) with a small effect size (ES=0.183). The post-hoc analysis shows that there were no significant post-intervention differences in PA scores between the three groups. However, there were significant differences between T0 and T1 follow up for the MFI group (p<0.001), T0 to T2 follow up and at T3 for the EX group (p<0.001) and between T0 and T3 for the CON group (p<0.001).

Body Mass Index (BMI)

At T0, T1, T2 time-points, there were no significant differences between groups for mean BMI scores; ( Table 3).

**Table 3.** BMI of overweight and obese adolescent between groups and within-group difference.

Time points	MFI Group (n=197) Mean±SD	EX group (n=199) Mean±SD	CON group (n=208) Mean±SD	Time Effect(T) F: ES: p value	Group Effect(G) F: ES: p value	Group x Time effect F: ES: p value
T0	23.1±1.99	23.2±2.04	23.0±1.74	233.687 0.237 < 0.001 *	7.790 0.005 < 0.001 *	4.934 0.013 < 0.001 *
T1	23.6±1.97	23.7±2.20	23.5±2.05			
T2	24.0±1.98	24.2±2.14	24.2±2.12			
T3	25.8±1.96	26.3±2.19	27.2±2.16			
	MFI group		EX group		CON group	
Time-points	Diff	p value	Diff	p value	Diff	p value
T0-T1	-0.469	1.000	-0.357	1.000	-0.506	1.000
T0- T2	-0.913	0.001 *	-0.935	<0.001 *	-1.161	<0.001 *
T0- T3	-2.657	<0.001 *	-3.074	<0.001 *	-4.17	< 0.001 *

* Significant difference p <0.05.

Analysis of the time and group effects ( Table 3) shows statistically significant changes in BMI over all the time points (p<0.001) with a small effect size (ES= 0.237). The group effect was also significant (p<0.001) but with a negligible ES. The group x time interaction also shows statistically significant interaction (p<0.001) but with a negligible ES. The post-hoc analysis shows that the differences in BMI from T0 to T1 were non-significant for all groups (p=1.000). From T0 to T1, there were significant increases for the MFI (p = 0.001), EX (p<0.001) and CON (p<0.001) groups. From T0 to T3, there were again significant increases (p<0.001) for each group. The biggest BMI increase during the study was in the CON group, with the MFI group increasing their mean score the least.

### 3.3 Secondary outcome measures

Health Related Quality of life (HRQOL).

In mean HRQoL scores; at T3, the MFI group showed highest change, followed by EX group and CON group had the least increase in HRQOL scores. The HRQOL values are presented in Table 4. The results show a statistically significant main effect of time (p<0.001) with small ES of 0.266; group effects were also significant (p<0.001) with a small ES (0.311). The time and group interaction (p<0.001) also showed a small ES of 0.234. From T0 to T1, both MFI and EX groups increased their mean HRQOL scores significantly (p<0.001), compared to the CON group (p = 1.000). At T2 and T3, the MFI and EX groups also increased scores compared to baseline values (p<0.001 for both groups) while the CON did not.

**Table 4.** Health related quality of life (HRQOL) score difference between groups and within-group difference.

Time points	MFI Group (n=197) Mean±SD	EX group (n=199) Mean±SD	CON group (n=208) Mean±SD	Time Effect(T) F: ES: p value	Group Effect(G) F: ES: p value	Group x Time effect F: ES: p value
T0	44.6±5.92	46.5±7.21	49.8±7.24	272.74, 0.266, <0.001 *	678.88, 0.311, <0.001 *	114.74, 0.234, <0.001 *
T1	56.2±6.92	49.6±7.26	50.5±7.49			
T2	65.4±7.65	54.1±7.70	51.0±5.12			
T3	69.0±5.13	54.1±6.80	50.7±7.44			
	MFI group		EX group		CON group	
Time-points	Diff	p value	Diff	p value	Diff	p value
T0-T1	-11.59	<0.001 *	-3.13	< 0.001 *	-0.76	1.000
T0- T2	-20.83	<0.001 *	-7.62	<0 .001 *	-1.23	1.000
T0- T3	-24.35	<0 .001 *	-7.59	<0 .001 *	-0.88	1.000

* Significant difference p<0.05.

## 4. Discussion

The primary objective of this study was to evaluate how a 12-week with long term follow-up multifactorial lifestyle intervention impacted PA levels and BMI in overweight and obese adolescents, with a secondary focus on changes in HRQOL. The adolescents who received the MFI intervention showed greater improvements in self-reported PA and HRQOL compared to those in EX and CON groups. The BMI increased in all the three groups, however MFI group showed least change. Furthermore, these changes were largely sustained by the MFI and EX groups for up to one year after the intervention.

The positive effects observed in PA levels are extremely important in the context of improving adolescent health and reducing the risk of chronic disease onset. ^38^^,^^39^ The changes could be credited to the supervised PA in the schools by PE teachers, ^40^ the long-term follow ups, ^41^ where teachers are in a unique position to motivate students and increase their self-awareness ^39^^,^^42^ plus the individual tailoring of exercise intensity using the PCERT guide. ^43^ Other factors that could contribute to the greater improvements in the MFI group include the educational and promotional component for students and families, ^44^^,^^45^ and the implementation of a theory-based intervention in addition to the PA intervention. ^31^ A review showed that school-based multifactorial interventions utilising PA promotional literature were linked positively to increased PA among adolescents. ^45^ This further emphasizes the need for schools to provide enough opportunities for students to participate in regular PA. Previous studies in the USA ^46^ and Australia ^47^ have also demonstrated similar findings, where school-based integrative, multiple behaviour interventions were found to be effective in improving PA levels among adolescents. The authors attributed lifestyle modifications to increasing awareness of the benefits of PA, increasing fruits and vegetable consumption, and involving parents and teachers in the interventions for adolescents.

We also found a slight and unexpected PA increase at the 12-month follow-up in the CON group. It is possible that some CON adolescents provided PAQ-A responses they believed the principal investigator expected, thus scoring higher in the questionnaire than was accurate. This is one of the potential drawbacks of using self-reported PA rather than direct measures. Repeated administration of questionnaires at multiple time points may have prompted students to realize their inactive lifestyle and change it, which is a positive behavioural change in the context of PA. This change could be because of the Hawthorne effect ^48^ or due to social desirability. ^49^ The Hawthorne effect refers to the tendency to change one’s behaviour in response to being observed or given attention by the researcher. ^50^^,^^51^ Several studies concluded that increases in PA among control group participants were attributed to social desirability, with participants providing responses that aligned with social expectations. ^52^^–^^54^ The participant-investigator interaction effect can neither be validated nor discounted in the present study.

In terms of BMI, the present study showed increases in all the three groups but with the MFI group showing the least increase. This could be due to increases in regular PA which reduced body fat as well as increasing relative lean muscle mass in the MFI and EX groups, thus a change in body composition. ^39^ As clinical indicators of weight gain, BMI classifications have limitations i.e. they do not take into account the proportions of fat mass and lean muscle mass components in total weight mass. ^55^ Therefore, BMI cut-off values may have high specificity in obesity diagnoses but low sensitivity to identify adiposity. ^56^

The multifaceted lifestyle approach of the intervention may also have contributed to positive BMI results for the MFI group. The intervention included parental support, dietary education on healthy eating with instructions and benefits of consuming higher quantities of vegetables and fruits while cutting down on calorie-dense food with culturally-appropriate illustrations. Also, the logbook embedded in the educational booklet plus regular participant follow-ups may have contributed to higher adherence to a healthy lifestyle among the MFI group. ^57^ These result align with a goal of the study to reduce negative energy imbalance among adolescents by encouraging better food choices and higher PA levels.

The perceived higher HRQOL in the MFI group compared to the EX and CON groups may be due to a variety of factors associated with the intervention, especially increased education, parental input and behaviour changes. HRQOL is influenced by sociodemographic and psychological factors, lifestyle choices, physical health, and family relationships. ^58^^–^^60^ Research indicates that children often adopt their parent’s lifestyle patterns, so it’s important for the parents to be educated and motivated to make healthy changes such as promoting healthy eating patterns, reducing screen time, demonstrating positive family dynamics through parental affection and autonomy promotion. ^58^^–^^60^ Along with parental education, regular PA involvement can have positive impact on various psychosocial factors like self-esteem, self-image, self-efficacy, social and peer interactions, and school performance. ^58^^–^^60^ Regular PA has also been associated with improved physical health parameters and reduced risk of chronic diseases. ^61^ These factors may directly or indirectly contribute towards better HRQOL among adolescents. However, our HRQOL findings are in contrast with studies by Resaland et al and Raj et al, where no change in HRQOL was observed among school-going participants. ^61^^,^^62^ The lack of change in these studies was concluded to be due to the interventions focusing only on PA for a short duration ^62^ and that the study population was treatment-seeking rather than being randomly allocated. ^58^ In our study, the long-term follow-ups assessing HRQOL and PA levels were extremely important to determine the sustainability of the multifactorial intervention benefits physically and psychologically.

### Study strengths and limitations

The study has strengths like cluster RCT design and long-term follow-ups for lifestyle changes. However, limitations include English medium school population sample, therefore results should be applied to government schools with caution, non-blinding of outcome measures and subjective assessment of PA due lack of funding and resources. Subjective measures like questionnaires can lead to over or under estimation of PA. To combat obesity, integrating lifestyle education, PA guidelines, and health promotion into school curriculums should be considered at policy-making levels. Evaluating policy-based interventions like replacing high-calorie food with healthier options should also be evaluated.

## Conclusion

The study suggests that a multifactorial lifestyle intervention, including education and regular exercise, is more effective in increasing physical activity levels and preventing BMI increases in overweight adolescents. Future studies with objective physical activity assessments into PA and lifestyle interventions are recommended.

## Ethics and consent

The study was conducted as per the principles of the Declaration of Helsinki and in accordance with the medical research involving human subject act (WMO). The current study is part of larger ongoing randomized trial. The study was approved by IRC-Institutional Research Committee (23/06/2018) and IEC- Institutional Ethics Committee of Kasturba Medical College and Kasturba Hospital Manipal (19/09/2018) (IEC: 536-2018). The clinical trial was registered in Clinical Trials Registry - India, Trial number CTRI/2019/04/018834, registered on 30/04/2019 ( https://www.ctri.nic.in/Clinicaltrials/pmaindet2.php?EncHid=MzE1Mzk=&Enc=&userName=CTRI/2019/04/018834). Permissions to approach the schools was sought from the Office of the Deputy Director of Public Instructions (DDPI), Udupi, Karnataka, India and each individual school principal’s permission was sought.

Each participant and their parents signed a written informed consent before participation. Participants received study objective, procedures and rights to withdraw any time during these study through participant information sheet. The study participation was entirely voluntary.

## Data Availability

Figshare: Adolescent PA, BMI and HRQOL. https://doi.org/10.6084/m9.figshare.26123824. ^64^ Data are available under the terms of the Creative Commons Attribution 4.0 International license (CC-BY 4.0). Figshare: CONSORT checklist for ‘Effect of a pragmatic lifestyle modification intervention on physical activity levels and body mass index among obese and overweight adolescents in Udupi, India: A cluster randomized trial’. https://doi.org/10.6084/m9.figshare.26124562. ^63^ Data are available under the terms of the Creative Commons Attribution 4.0 International license (CC-BY 4.0). Harvard Dataverse: Replication Data for: Fig 1 Consort flowchart of the study. https://doi.org/10.7910/DVN/ADFWTX. ^65^ Data are available under the terms of the Creative Commons Zero “No rights reserved” data waiver (CC0 1.0 Public domain dedication).
