# Supplementary material for: Effect of a pragmatic lifestyle modification intervention on physical activity levels and body mass index among obese and overweight adolescents in Udupi, India: a cluster randomized trial
Source: F1000Res. 2024 Aug 1;13:859. [Version 1] doi: 10.12688/f1000research.153483.1 (PMC11303949; doi:10.12688/f1000research.153483.1)
Supplement: Peer review history

# Reviewer report: Reviewer response for version 1

**DOI:** 10.5256/f1000research.168393.r310428

**Published:** 2024-08-06

**Authors:** Jaya Shanker Tedla

**Affiliations:** 1 Department of Medical Rehabilitation Sciences, College of Applied Medical Sciences, King Khalid University, Abha, Aseer Province, Saudi Arabia

As a reviewer of this paper, I appreciate the multifactorial approach adopted by the authors to see the effects on school children. The effect sizes reported in this study can guide future research by providing benchmarks for expected changes. Researchers can use these benchmarks to design studies with adequate power to detect meaningful effects. However, there are few clarifications required.

 1) The intervention is described as culturally appropriate. Elaborating on how it was tailored to fit the participants' cultural context would provide valuable insights for replicating the study in other settings.

 2) A more detailed comparison with similar studies conducted in different countries or regions could benefit the discussion. This would help contextualize the findings and highlight the unique aspects of the intervention.

 3) Suggesting specific areas for future research, such as exploring the long-term impact of the intervention on different age groups or in different settings, would be beneficial for advancing the field.

Is the work clearly and accurately presented and does it cite the current literature?

Yes

If applicable, is the statistical analysis and its interpretation appropriate?

Yes

Are all the source data underlying the results available to ensure full reproducibility?

Yes

Is the study design appropriate and is the work technically sound?

Yes

Are the conclusions drawn adequately supported by the results?

Yes

Are sufficient details of methods and analysis provided to allow replication by others?

Yes

Reviewer Expertise:

Physical Therapy

I confirm that I have read this submission and believe that I have an appropriate level of expertise to confirm that it is of an acceptable scientific standard.

# Reviewer report: Reviewer response for version 1

**DOI:** 10.5256/f1000research.168393.r310425

**Published:** 2024-08-05

**Authors:** Snehal Subrat Samal

**Affiliations:** 1 Datta Meghe Institute of Higher Education & Research, Wardha, India

I would like to thank the journal for providing me with the opportunity to review the article. The article articulates very nicely that it is the need of the hour for adolescent obesity intervention in India. There are a few suggestions for making the study better:

 1) The study mentions the importance of parental involvement in lifestyle modifications. Still, providing more detailed information on how parents were engaged and the specific activities they participated in would be beneficial.

 2) While the study includes long-term follow-ups, more details on the frequency and nature of these follow-ups would help understand their impact on the sustainability of the intervention benefits.

 3) The educational and promotional components for students and families are mentioned as contributing factors to the success of the intervention. Providing more specifics about the content and delivery methods of these educational materials would enhance the understanding of their effectiveness

Is the work clearly and accurately presented and does it cite the current literature?

Yes

If applicable, is the statistical analysis and its interpretation appropriate?

Yes

Are all the source data underlying the results available to ensure full reproducibility?

Yes

Is the study design appropriate and is the work technically sound?

Yes

Are the conclusions drawn adequately supported by the results?

Yes

Are sufficient details of methods and analysis provided to allow replication by others?

Yes

Reviewer Expertise:

Neurophysiothearpy, Musculoskeletal Physiotherapy

I confirm that I have read this submission and believe that I have an appropriate level of expertise to confirm that it is of an acceptable scientific standard.
